# Free and bound excitonic effects in Al_0.5_Ga_0.5_N/Al_0.35_Ga_0.65_N MQWs with different Si-doping levels in the well layers

**DOI:** 10.1038/srep13046

**Published:** 2015-08-12

**Authors:** Chenguang He, Zhixin Qin, Fujun Xu, Mengjun Hou, Shan Zhang, Lisheng Zhang, Xinqiang Wang, Weikun Ge, Bo Shen

**Affiliations:** 1 State Key Laboratory of Artificial Microstructure and Mesoscopic Physics, School of Physics, Peking University, Beijing 100871, China; 2 Collaboration Innovation Center of Quantum Matter, Beijing 100084, China

## Abstract

Free exciton (FX) and bound exciton (BX) in Al_0.5_Ga_0.5_N/Al_0.35_Ga_0.65_N multiple quantum wells (MQWs) with different Si-doping levels in the well layers are investigated by photoluminescence (PL) spectra. Low temperature (10 K) PL spectra identify a large binding energy of 87.4 meV for the BX in undoped sample, and 63.6 meV for the BX in Si-doped (2 × 10^18 ^cm^−3^) sample. They are attributed to O-bound and Si-bound excitons, respectively. The large binding energies of BX are assumed to originate from the strong quantum confinement in the quantum wells, which also leads to a stronger FX PL peak intensity in comparison with BX at 10 K. Si-doping is found to suppress the FX quenching by reducing threading dislocation density (TDD) in the well layers, leading to a significant improvement of IQE from 33.7% to 45%.

In the past decade, AlGaN-based deep ultraviolet light-emitting diodes (DUV-LEDs) have attracted considerable attentions owing to their wide application potentials in biomedical and analytical instrumentation, sterilization and decontamination, UV curing, and high density optical recording^1^,^2^. Compared with conventional mercury vapor lamps, AlGaN-based solid-state UV sources have significant advantages in terms of size, operation voltage, spectral tunability, and being environmentally friendly. Impressive research efforts have achieved luminescence from 210 nm to 365 nm over the range of full Al compositions^1^,^2^,^3^,^4^. Despite these tremendous opportunities and progress, DUV-LEDs still suffer from relatively low external quantum efficiencies (EQEs) as well as output light power, in comparison with commercial blue LEDs. The EQEs of DUV-LEDs with wavelength shorter than 280 nm are less than 11% in previous reports^1^,^2^,^4^,^5^. As a result, the output power of DUV-LEDs with corresponding wavelength is still under 100 mW up to date^1^,^2^,^6^.

IQE is the key factor determining the performance of DUV-LEDs. It is known that higher IQE can be achieved by using a better underlying epilayer having low TDD^7^. The IQE can also be improved through optimization of the MQWs structures^8^,^9^,^10^. A detailed knowledge of the exciton behavior in high-Al-content AlGaN/AlGaN MQWs would certainly provide useful guidance for MQWs structure improvement. Along that direction, exciton behavior in GaN bulk has been investigated by many groups. The binding energy of FX, donor-bound exciton (DX), and acceptor-bound exciton (AX) in GaN has been reported in large volumes^11^,^12^,^13^. The radiative recombination lifetime of FX and BX in GaN ranges from 125 ps to 530 ps^13^,^14^. For bulk AlN, the binding energy of FX has also been reported, ranging from 47 meV to 80 meV^15^,^16^,^17^. As for AlGaN/GaN and InGaN/GaN MQWs, multiple factors which affect the FX and BX binding energy have been studied by theoretical calculations^18^,^19^. With regard to high-Al-content AlGaN bulk and AlGaN/AlGaN MQWs, many reports focus on the exciton localization behavior and optical polarization properties, while few reports pay attention to the relevant parameters of FX and BX. In this study, we investigated the FX and BX behavior of high-Al-content AlGaN/AlGaN MQWs with different Si-doping levels in the well layers.

Three MQWs samples (sample-A, -B, and -C) were prepared, whose structures are shown in Fig. 1. The active region consists of five 2.5-nm-thick Al_0.35_Ga_0.65_N well layers separated by 10-nm-thick Al_0.5_Ga_0.5_N barrier layers. The only difference among sample-A, -B, and -C lies in the Si-doping concentration of the well layers, being 0, 5 × 10^17^, and 2 × 10^18^ cm^−3^, respectively.

A collection of near-band-edge time-integrated PL spectra at 10 K is presented in Fig. 2 (a). It shows that two distinct peaks at 275.40 nm and 280.85 nm appear in the spectrum of sample-A. Similarly, two peaks at 276.72 nm and 280.70 nm are observed from the spectrum of sample-C. It is clear that the peak spacing between the FX and BX peak is different for sample-A and -C. We attribute the two peaks in either sample-A or sample-C to FX and BX luminescence from Al_0.5_Ga_0.5_N/Al_0.35_Ga_0.65_N MQWs. The energy spacing between the FX and BX peak represents the binding energy of a “certain impurity or defect”-bound exciton. Two BX binding energies of 87.4 (for sample-A) and 63.6 meV (for sample-C) can then be derived from the PL spectra. While for sample-B, besides the FX peak at 276.46 nm, there are two identifiable BX peaks on the long-wavelength side, whose corresponding binding energies are 89.1 and 63.6 meV, respectively.

The binding energy difference for BX in undoped sample-A and Si-doped sample-C is attributed to the fact that the excitons are bound to different impurity atoms or defects in the two cases. In nominally undoped and Si-doped AlGaN, n-type conductivity is generally found^20^. It is thus suggested that the BX in our samples are most likely to be donor-bound excitons (DX). It has been well established that the most common donors in undoped AlGaN are O_N_ (O substitutes the nitrogen site) and V_N_ (nitrogen vacancy). Furthermore, O_N_ is more easily to form compared with V_N_ because of its relatively low formation energy^20^. As such, O_N_ is usually the dominant donor in unintentionally-doped AlGaN. In the case of Si-doped AlGaN, however, Si_Ga_ (Si substitutes the Ga site) will form prior to O_N_ because of an even lower formation energy^20^. In addition, Si is also found to suppress the incorporation of O by formation of Si-O bond^21^. As a result, the concentration of O_N_ will decrease gradually as Si-doping concentration increases. Therefore, DX1 in sample-A and DX2 in sample-C are attributed to O-bound exciton and Si-bound exciton, respectively. For the medium-level doped sample-B, O_N_ cannot be suppressed completely, two types of DX respectively appeared in sample-A (DX1) and sample-C (DX2) are hence both observable.

As mentioned above, the binding energy of O-bound exciton in sample-A and Si-bound exciton in sample-C is 87.4 and 63.6 meV, respectively. Such large DX binding energies have rarely been reported in III-nitrides system to our knowledge. The typical values of that for BX in GaN only vary from 6 to 25 meV^12^,^13^. Generally, the DX binding energy can be empirically estimated through Haynes’s rule:


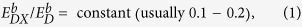


where 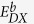
and 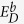
are the binding energies of the donor-bound exciton and donor itself, respectively. It has been reported that the binding energies of Si_Ga_ 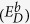 and O_N_ 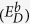 in GaN are 30.18 and 33.20 meV, respectively^12^. The derived binding energies of excitons bound to Si_Ga_ 
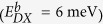
 and O_N_ 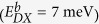 in GaN followed the empirical Haynes’s rule well with a constant of 0.214. As for AlN, Neuschl *et al.* found that the binding energy of Si-bound exciton 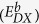 was 28.5 meV^22^. They estimated a value of 133 meV for the binding energy of Si donor 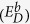, assuming the same constant of 0.214. But it is more generally accepted that the binding energy of O_N_ 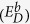 and Si_Ga_ 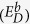 in AlN is about 340 and 240 meV, respectively^23^,^24^. If so, the binding energy of Si-bound exciton 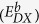 and O-bound exciton 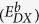 in AlN should be 73 and 51 meV, respectively. Using a linear interpolation approximation, the binding energy of O-bound exciton 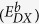 and Si-bound exciton 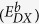 in bulk Al_0.35_Ga_0.65_N is 30.1 meV and 21.8 meV, respectively. However, the corresponding values in our MQWs samples reach up to 87.4 and 63.6 meV, respectively, about 2.9 times of that in bulk Al_0.35_Ga_0.65_N. It demonstrates that the strong quantum confinement in MQWs indeed leads to a great enhancement of the DX binding energy, which is consistent with the calculation for a finite potential well^25^.

The PL spectrum from each of our samples shows that the peak intensity of the FX luminescence is stronger than that of BX luminescence. However, in the case of bulk GaN, it has been reported that the PL spectra are usually dominated by BX luminescence at low temperature when FX and BX are observed simultaneously^12^,^13^,^14^. It is suggested that this discrepancy in peak intensity ratio originates from the difference in the density and oscillator strength of FX and BX, as discussed below.

First, FX has two main channels to relax at 10 K: radiative recombination and to be captured by impurities or defects forming DX. Therefore, the density of FX and BX is decided by these two competitive processes, which are characterized by FX radiative recombination lifetime *τ*_*r*_ and FX capture time *τ*_*tr*_, respectively. The FX capture time can be expressed as:


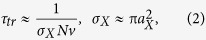


where 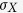
is the capture cross-section, *N* is trap concentration, *v* is the thermal velocity of FX diffusion, and *a*_*X*_ is the Bohr radius of FX. In the case of GaN, it is reported that *τ*_*r*_ and *τ*_*tr*_ are 375 and 16 ps at 4 K, respectively^11^. That implies that the chance of FX luminescence is smaller than FX capture, which is in favor of BX formation. For bulk AlGaN, the Bohr radius *a*_*X*_ becomes smaller than that of GaN^26^. Moreover, we have to consider that in the case of AlGaN/AlGaN MQWs, *a*_*X*_ will decrease sharply because of being compressed along the growth direction. A smaller *a*_*X*_ represents a larger overlap of the electron and hole wavefunction for FX radiative recombination process as well as a smaller capture cross-section *σ*_X_ for FX capture process. This leads to a shorter FX lifetime *τ*_*r*_ and a longer capture time *τ*_*tr*_, resulting in a decrease of the density of BX^27^.

Second, the oscillator strength of FX and BX is proportional to 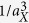 and 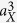, respectively^28^,^29^. As mentioned above, the Bohr radius *a*_*X*_ of AlGaN in MQWs decreases sharply compared with that in GaN, leading to a salient increase in the oscillator strength of FX and a sharp decrease in the oscillator strength of BX. As a result, the transition probability of FX increases, at the expense of that of BX.

Under the combined influence of density and oscillator strength, the peak intensity of the FX luminescence obviously exceeds that of the BX luminescence for our MQWs samples. It is worth noting that the above analysis about the Bohr radius neglected the quantum-confined Stark effect (QCSE), because the large band offset and narrow well width in our samples will result in a high energy level above the triangular potential wells, meaning that the QCSE is very weak^30^.

Excitation-power-dependent PL was employed to confirm the nature of the FX and BX peaks, as presented in Fig. 2(b). The dependence of the integrated PL intensity of the short-wavelength-side peaks on the excitation-energy density gives approximately I ∝ P, indicating that the luminescence results from FX recombination. While the integrated PL intensity of the long-wavelength-side peaks increase sublinearly with the excitation power. The power exponent is 0.88 for sample-A and 0.87 for sample-C, respectively. This is due to the saturation of the BX radiative recombination process, which results from the finite donor concentration. For sample-B, DX1 peak and DX2 peak are hard to distinguish because they are too close to each other. Only the dependence of their total integrated PL intensity on the excitation-energy density can be obtained with a power exponent of 0.76. All these results support our peak recognition.

Figure 3 (a)–(c) show temperature-dependent PL spectra from 10 K to 300 K. The FX and DX in all samples show obvious PL intensity decrease with temperature. In particular, the DX intensities show faster decrease than those of FX as temperature increases. The internal quantum efficiency (IQE), estimated as the integrated PL intensity at room temperature divided by that at 10 K, is 33.7%, 38.2%, and 45% for sample-A, sample-B, and sample-C, respectively. The different temperature characteristics of DX and FX are attributed to different thermal activated processes, as discussed below.

The decrease of the DX PL intensity is mainly due to DX dissociation, which can be described by:


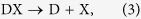


where D and X refer to donor and free exciton, respectively. The activation energy of the DX dissociation is approximately equal to the binding energy. On account of the large binding energies of DX1 (87.4 meV) and DX2 (63.6 meV), the DX peaks can be well identified even at 160 K in sample-A and 130 K in sample-B and -C. It is worth mentioning that DX1 and DX2 luminescence can be seen in almost all the samples at intermediate temperatures. The explanation of the fact is that there should be tiny amounts of Si residue in the reactor chamber after n-AlGaN growth. In addition, O contamination can never be completely avoided for MOCVD growth. Therefore, Si-related and O-related excitons should exist at the same time. Only at low temperature, the donor-bound exciton luminescence associated with the higher-concentration one should be dominant. The lower-concentration-donor-bound exciton luminescence would be covered up because the spacing between DX1 and DX2 are too close. At intermediate temperatures, however, DX1 and DX2 luminescence could both be observed with similar but weak intensity.

The decay of the FX PL intensity is usually caused by more than one nonradiative recombination channels, but usually dominated by a high-activation-energy process. For understanding of the FX quenching mechanisms, the FX integrated PL intensities from 10 to 300 K in Fig. 4 (dots) are analyzed by Arrhenius plots fitting (lines). Assuming that there are two dominated thermally activated processes, the temperature-dependent FX integrated PL intensity *I(T)* can be modeled by:


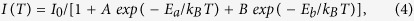


where *I*_*0*_ is the FX integrated PL intensity at 10 K, A and B are rate constants, *E*_*a*_ and *E*_*b*_ are the activation energies of the nonradiative recombination processes, and *k*_*B*_ is the Boltzmanm’s constant^31^. This expression provides a very good fit to our experimental datas with *E*_*a*_ being 9, 12, and 15 meV for sample-A, -B, and -C, respectively. Simultaneously, *E*_*b*_ is 100, 105, and 120 meV for sample-A, -B, and -C, respectively. The detailed results are displayed in Table 1. The first quenching mechanism (characterized by *E*_*a*_) is attributed to the delocalization of excitons from potential fluctuations in the AlGaN well layers. It has been reported that a thickness fluctuation of 1 monolayer in a narrow well layer will make a significant difference to the confinement energies^32^. The thickness of our well layers is as narrow as about 10 monolayers, therefore, the potential variations in the well layers should be dominated by well/barrier interface roughness. The localization energy caused by interface roughness in our samples is obviously small, implying an abrupt MQWs interface. The value of *E*_*b*_ increases with Si-doping level. This may result from suppression of the nonradiative recombination which is generally related to threading dislocation. Wet etching techniques are used to check that. As shown in Fig. 5, AFM images show a remarkable reduction of TDD with increase of the Si-doping concentration, in good agreement with our speculation. So we hold the view that a reduced TDD caused by Si-doping leads to a higher activation energy for FX quenching, thus an increased IQE.

In summary, low temperature PL spectra (10 K) of Al_0.5_Ga_0.5_N/Al_0.35_Ga_0.65_N MQWs identify a binding energy of 87.4 meV for BX in undoped sample, and 63.6 meV for BX in Si-doped (2 × 10^18 ^cm^−3^) sample, referring to O-bound exciton and Si-bound exciton, respectively. The large binding energies of BX are 2.9 times of that in bulk Al_0.35_Ga_0.65_N estimated by a linear interpolation approximation. This significant increase in binding energies is assumed to originate from the strong quantum confinement in MQWs.

Much higher FX PL peak intensity in comparison with BX is suggested to result from a smaller Bohr radius of FX in Al_0.5_Ga_0.5_N/Al_0.35_Ga_0.65_N MQWs. Si-doping are found to suppress the FX quenching by reducing TDD in the well layers, leading to a significant improvement of IQE from 33.7% to 45%.

## Methods

### Samples Preparation

**Figure 1:**
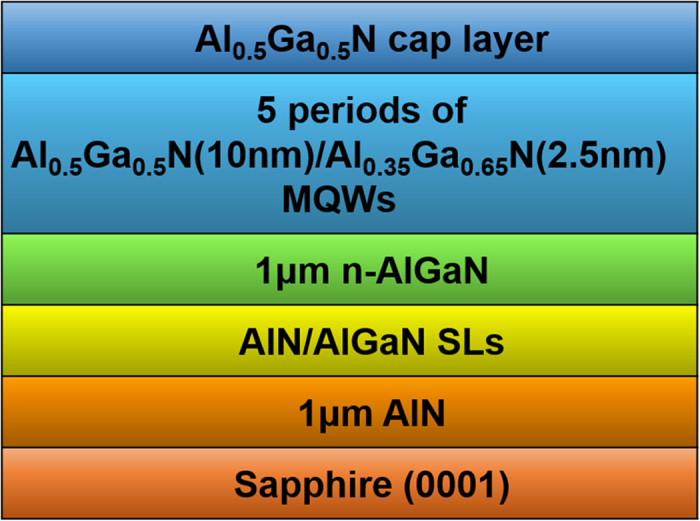
Schematic illustration of the MQWs structures.

In this study, three MQWs samples (sample-A, -B, and -C) were grown on 2-in. (0001) sapphire substrates by low pressure metal organic chemical vapor deposition (LP-MOCVD). Deposition was initiated from a 1-μm-thick AlN layer, followed by a multi-period AlN/AlGaN superlattice (SL) layer and a 1-μm-thick Si-doped AlGaN layer. The active region consists of five 2.5-nm-thick Al_0.35_Ga_0.65_N well layers separated by 10-nm-thick Al_0.5_Ga_0.5_N barrier layers. Finally, the structure was completed with a 25-nm-thick Al_0.5_Ga_0.5_N cap layer. A cross section schematic of the MQWs samples is shown in Fig. 1.

### Optical and Surface Morphology Measurements

A 4th harmonic of Q-switched YAG:Nd laser (λ = 266 nm, pulse width = 7 ns) was used for PL excitation, and an Ocean Optics USB2000+VIS-NIR fiber optic spectrometer was employed to record the PL spectra. A closed-cycle helium cryosystem provided the variation of temperature in a range from 10 K to 300 K. Excitation power tuning was realized by THORLABS NUK01 neutral density filters. The surface morphology for sample-A, -B, and -C after molten KOH etching was characterized by Bruker Dimension ICON-PT atomic force microscopy (AFM).

## Additional Information

**How to cite this article**: He, C. *et al.* Free and bound excitonic effects in Al_0.5_Ga_0.5_N/Al_0.35_Ga_0.65_N MQWs with different Si-doping levels in the well layers. *Sci. Rep.* **5**, 13046; doi: 10.1038/srep13046 (2015).

## Figures and Tables

**Figure 2:**
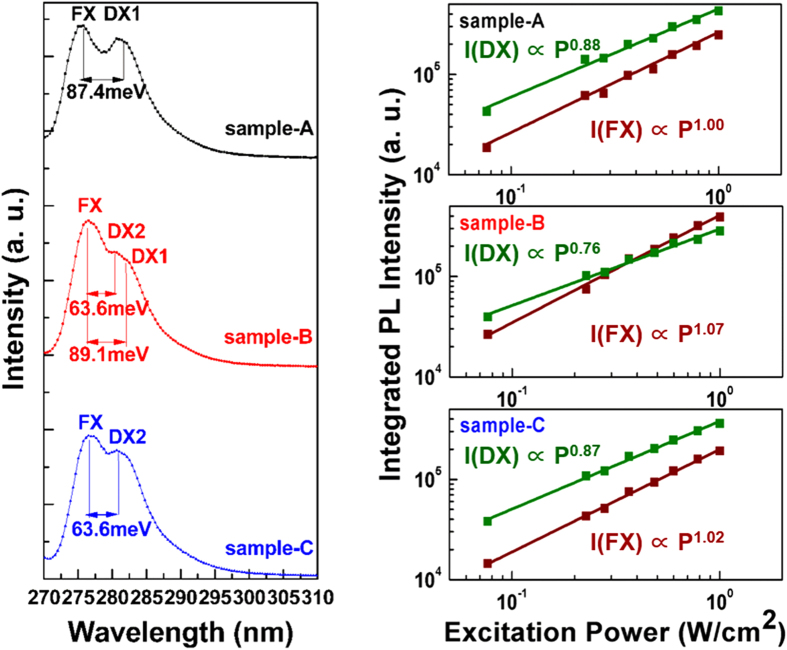
(**a**) Time-integrated PL spectra at 10 K for all samples. (**b**) The dependence of integrated PL intensity on excitation-energy density at 10 K for all samples.

**Figure 3:**
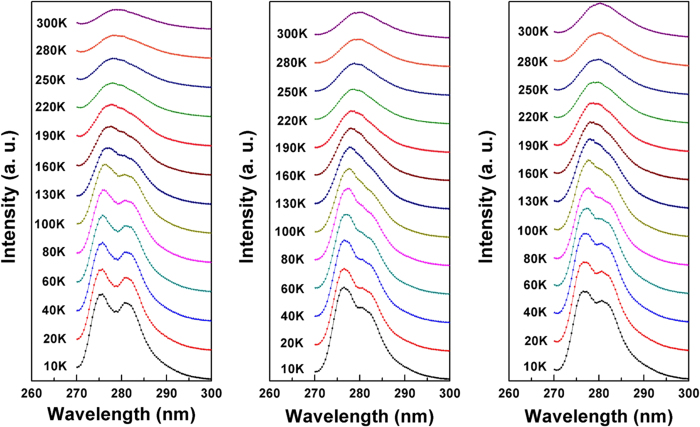
(**a**)–(**c**) Time-integrated PL spectra from 10 K to 300 K for sample-A, -B, and -C, respectively.

**Figure 4:**
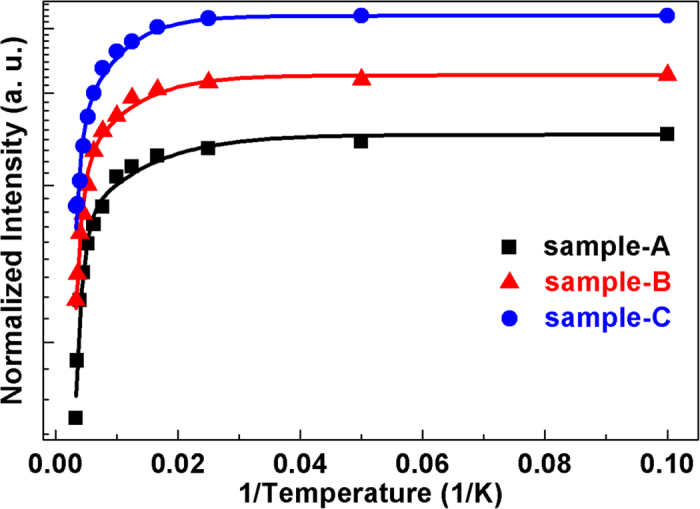
FX integrated PL intensity (dots) and Arrhenius plots fitting (lines) for sample-A, -B, and -C, respectively.

**Figure 5:**
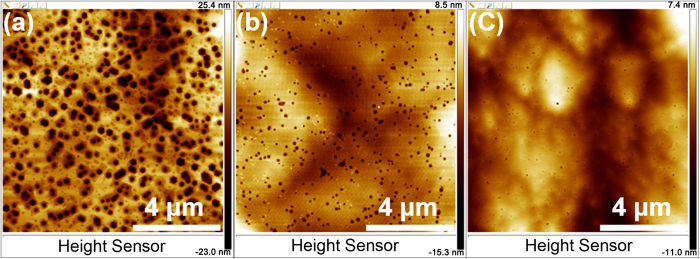
(**a**)–(**c**) AFM images for sample-A, -B, -C after molten KOH etching under the same conditions for 4 minutes, respectively.

**Table 1:** The fitted results with respect to the experimental temperature-dependent PL integrated intensities for sample-A, -B, and -C.

	A	E_a_ (meV)	B	E_b_ (meV)
Sample-A	0.69	9	77.62	100
Sample-B	0.85	12	70.58	105
Sample-C	1.14	15	91.22	120
